# A review of the response to HIV/AIDS in Trinidad and Tobago: 1983–2010

**DOI:** 10.1080/17290376.2013.869406

**Published:** 2014-01-03

**Authors:** Christine Laptiste, Vyjanti Beharry, Patricia Edwards-Wescott

**Affiliations:** a BSc Economics, MSc Economics, is a Research Fellow at the HEU, Centre for Health Economics, The University of the West Indies, St. Augustine, Trinidad; b BSc Economics, MPhil., Economics, is a Junior Research Fellow at the HEU, Centre for Health Economics, The University of the West Indies, St. Augustine, Trinidad; c BSc Economics, MSc Economics, is a Junior Research Fellow at the HEU, Centre for Health Economics, The University of the West Indies, St. Augustine, Trinidad

**Keywords:** HIV/AIDS, response, health sector, treatment and care, prevention, VIH/SIDA, riposte, secteur de la santé, traitement et soins, prévention

## Abstract

This paper examines the character of the response to HIV/AIDS in Trinidad and Tobago and assesses the impact of the response on reducing the spread of the epidemic. The launch of the National HIV/AIDS Strategic Plan in 2004 signalled the intent of the government to take the response to HIV/AIDS to a different level. This is seen by the sheer increase in the volume of resources allocated to the response from the levels of the 1980s and 1990s. The expectation was that there would be increased cohesiveness, which would allow for targeted interventions to be more effective. Though in 2009, there was a slight increase in the HIV prevalence rate to 1.5%, this was due mainly to improvements in access to antiretrovirals and same-day testing as well as improvements in data collection and analysis. The annual number of new infections fell from a high of 1709 in 2003 to 1154 in 2010. Additionally, great strides have been made in the prevention of mother-to-child transmission programme with some regions reporting 100% coverage of antenatal attendees. The study indicates that the country has responded relatively well in the areas of Strategic Planning, Care and Support, and Prevention and there has been involvement by both the public and private sector (NGOs in particular), in the response. However, there are gaps in the provision of social services and the implementing legislation to protect the rights of persons living with HIV/AIDS. Of note is the fact that a successful response to the HIV/AIDS epidemic is one that embraces all social groups, all spheres of activity and all areas of the country.

## Abstract (FR)

Le présent document examine la nature de la riposte au VIH/SIDA à la Trinidad et Tobago, et évalue l'impact de cette reaction sur la reduction de la propagation de l'épidémie. Le lancement du Plan Stratégique National sur le VIH/SIDA (NSP) en 2004 a marque l'intention du gouvernement de prendre la riposte au VIH/SIDA à un niveau différent. Ceci est mis en evidence par la simple augmentation du volume des resources allouées à cette riposte par rapport au niveau des années 1980 et 1990. L'objectif etait une cohesion meilleure, ce qui permettrait des interventions ciblées en vue d' être plus efficace. Bien qu'en 2009 il y a eu une légère augmentation poussant le taux de prévalence du VIH à 1,5%, ceci était du, pour l'essentiel, à l'amélioration de l'accès aux antirétroviraux (ARV), aux tests à resultat immediat, ainsi qu' à des ameliorations dans la collecte et l'analyse des données. Le nombre annuel de nouvelles infections est passé du maximum de 1 709 en 2003 à 1 154 en 2010. En outre, d'enormes progres ont été realizes dans le programme de prévention de transmission màre-enfant (PTME) avec certaines régions reportant 100% de couverture des participants aux soins prénataux. L'étude indique que le pays a réagi relativement bien dans les domaines de planification stratégique, de soins et appui, et de prevention, et, il y a eu un bonne participation a cette riposte par les secteurs public et privé, en particulier les ONG. Cependant, il existe des lacunes dans la prestation de services sociaux et dans la législation pour protéger les droits des personnes vivant avec le VIH/SIDA (PVS). Il est a noter que l'efficacité de la riposte à l'épidémie de VIH/SIDA englobe tous les groups sociaux, toutes les spheres d'activité et toutes les régions du pays.

## Introduction

In a region where the total population of the English-speaking countries is less than eight million and where some national populations are less than 100,000, the continued presence of HIV/AIDS can have a negative impact on development efforts. However, regionally there are encouraging signs that fewer people are becoming newly infected and dying from AIDS-related causes (WHO/UNAIDS/UNICEF 2011). Still, there is a call for a transformation to more effective and targeted responses backed by the requisite financial and human resource investment in order to achieve higher positive impacts (UNAIDS 2011).

In Trinidad and Tobago, since the first diagnosis of eight cases of HIV in 1983, the cumulative number of new HIV-positive cases was reported at 22,829 by the end of 2010. Further, the number of AIDS cases and AIDS-related deaths had climbed to 6407 and 3999, respectively in 2010, based on public sector reporting (Republic of Trinidad and Tobago 2013). Available data point to the fact that though the epidemic is generalized, there are pockets of high concentration in some population groups.

With strong political support, active involvement of faith-based organizations, decentralized programmes and confidential voluntary counselling and testing (VCT), early successes in Uganda's fight against the epidemic influenced the structure of response programmes in other countries (USAID 2002). Trinidad and Tobago adopted the ‘Three Ones’ principle (UNAIDS 2005) to respond to the HIV/AIDS epidemic, which are:

- One national AIDS coordinating authority with a broad-based multisectoral mandate
- One AIDS action framework to coordinate the work of all partners
- One national monitoring and evaluation system (NACC 2010a).

The government's initial response was grounded in the Ministry of Health and occurred at the time when prevention was the accepted wisdom on how to deal with the disease. Although HIV/AIDS is not solely a public health issue, the health sector's response is vital to any initiative aimed at reducing its spread, as this sector bears heavy responsibility for treatment, care and monitoring activities. It is in this regard that the configuration of the health sector – the interaction of its technical and systemic components – is critical to reducing incidence, morbidity and mortality associated with HIV/AIDS.

In 1983 a National Surveillance Committee was created under the aegis of the Ministry of Health. This committee was charged with the responsibility of information gathering, surveillance, ensuring safe blood transfusion systems, and public education about HIV/AIDS itself as well as prevention methods. Meanwhile, in 1986 a Tobago AIDS Committee was formed and was succeeded in 1988 by the Tobago Hospital AIDS Committee. The main focus of this committee was the placement of patients at hospital.

The National HIV/AIDS Strategic Plan (NSP) was launched in March 2004. This was the instrument for initiating the required expanded response to HIV/AIDS. Along with the launch of the NSP was the establishment of the National AIDS Coordination Committee (NACC), which had the mandate of implementing the NSP. Essentially, the NACC was created to pull together the disparate efforts of government, private sector, workplace, community, civil society and individuals. Catering to the specific needs of Tobago, the Tobago HIV/AIDS Coordinating Committee (THACC) was also established and operated under the ambit of the Tobago House of Assembly. The THACC had representation on the NACC. Due to implementation problems, the outer time bound of the NSP was extended from 2008 to 2010.

## Methods

Several technical and non-technical reports produced by the NACC and other agencies were reviewed and a general literature search was also conducted. Newsletters from Regional Health Authorities (RHAs) and unpublished reports from agencies involved in the fight against HIV/AIDS as well as legal documents were also perused. Additionally, discussions were held with key stakeholders located in various institutions. Most of the quantitative data were derived from technical reports of the NACC.

## The public health sector

### National AIDS Committee

In 1987, the National AIDS Committee (NAC) was created by the Cabinet and had the responsibility for policy formulation, programme monitoring and evaluation. The National AIDS Programme (NAP) functioned as its administrative arm. By June 1989, there were six subcommittees of the NAC, namely:

- Research and surveillance;
- Public education;
- Non-governmental organizations;
- Care and support;
- Legal and ethical; and
- Training.

The NAP carried out the policies and programmes of the various subcommittees, especially in the areas of information, education and communication. One of the major re-configurations of the NAP and NAC occurred in 1998, when the UN Theme Group on HIV/AIDS developed a proposal for the restructuring of the NAP and NAC under what became known as the National AIDS Programme Co-ordinating Unit (NAPCU). The UN Theme Group on HIV/AIDS, in facilitating UN's response to the epidemic in Trinidad and Tobago, assessed the activities of other agencies with a view to mainstreaming HIV into the country's social and economic programmes. It was also involved in resource mobilization and advocacy in the carrying out of HIV/AIDS programmes and made a key contribution in the formation of the NAPCU (HEU 2001).

In 2006 there was yet another restructuring and renaming to the HIV and AIDS Coordinating Unit (HACU), which was designed to be policy oriented. The HACU also had responsibility for funding as well as regulating, setting standards and coordinating the programmes of the five RHAs (Ministry of Health 2012).

Under the NAP, there were several initiatives. One of the main ones was Rap Port, a youth drop-in centre targeting the age group 13 – 25 years, created in response to the epidemic of HIV/AIDS/STIs affecting the youth population. The primary goal of Rap Port is ‘to promote healthy lifestyle practices among the youth by creating a supportive environment that would engender information flow, education, communication and counselling referral services’ (Joseph 1999:7). Activities undertaken by this arm of the NAP include self-development programmes; counselling; behaviour change communication projects using lectures, video, theatres and rap sessions; and an outreach programme through the electronic media. With its dynamic delivery style, Rap Port receives excellent feedback from youth. During the period 1995 – 1999, approximately 5678 youths accessed the in-house programmes with an additional 158,940 utilizing the outreach activities (HEU 2001). This initiative enjoyed success and grew to include three drop-in centres located in the Port of Spain, San Fernando and Arima.

In October 2010, each drop-in centre of the Rap Port programme was re-assigned to operate under the auspice of the area RHA – Port of Spain, North West RHA; San Fernando, South-West RHA (SWRHA) and Arima, North Central Regional Health Authority (NCHR) (Rap Port 2012). The programme continues to make an impact with, in addition to other activities, its four main programmes (i) Carnival Season – an open period to conduct sessions in various organizations, (ii) Rap Program – conducted for post-Secondary Entrance Assessment (SEA) students going into secondary school – after SEA, (iii) Rap Camp – conducted during the July August vacation for students on vacation and (iv) World Aids Day Activities conducted during the November to December period (Rap Port 2012).

Another important initiative, which has its beginnings in the NAP but is an independent NGO, is the National AIDS Hotline. Staffed by volunteers, this facility provides confidential and anonymous listening to persons living with HIV/AIDS (PLWHA) or persons affected by issues related to HIV/AIDS. The volunteers are carefully screened and are initially exposed to a 40-hour training programme comprising core information about the disease, basic knowledge of human behaviour, behavioural and emotional responses to the diagnosis, issues of death and dying, listening and counselling skills. Partially funded by the Ministry of Health, the Hotline is run by a board of management, and statistical data are compiled for submission to the Ministry of Health, HACU, CAREC, Joint United Nations Programme on HIV/AIDS (UNAIDS) and Pan American Health Organization (PAHO) (National AIDS Hotline 2012).

### The Queen's Park Counselling Centre and Clinic

Through its varied health sector institutions, the state has been providing services in the areas of prevention, diagnostics, counselling, treatment and monitoring. Initially established to provide counselling and clinical care for clients presenting with STIs, the Queen's Park Counselling Centre and Clinic (QPCC&C) was subsequently given the responsibility to serve as the main referral centre for persons who were found to be HIV positive and required counselling (Yearwood & Beharry 2003).

Concerns raised by the PLWHA community about QPCC&C indicate that breaches of confidentiality and lack of professionalism by the healthcare workers were problems. Further, the need for privacy at the Centre was also highlighted. In addition, due to its specialized character as an HIV/AIDS/STI clinic, it became a place that was largely shunned by the public. Many persons did not want to be seen at the clinic for fear of being marked with the stigma attached to the clinic.

One way of addressing the stigma concerns was the integrating of the QPCC&C field services with Antenatal and Sexually Transmitted Diseases Clinics in health centres in Arima, San Fernando, Sangre Grande, Tobago, Couva, Chaguanas, Rio Claro, Princes Town and Siparia (GoRTT 2012).

### Public hospitals

All six secondary health institutions that are strategically located in each of the five health regions, deliver generalized palliative care and treatment for all opportunistic infections (Yearwood & Cumberbatch 2003). PLWHA with suspected TB are referred to the Chest Clinic at the Caura Hospital. In 2009, 31% of tested TB cases were HIV positive (NACC 2010a). There have been reports however, that members of staff at health institutions are apprehensive when caring for HIV/AIDS clients, as is evident by the use of unnecessary infection control practices (KPMG 2001; NACC 2010a).

Ward 2 at the San Fernando General Hospital is a unit that provides specialized care for AIDS patients – an initiative spearheaded by the late Dr Edward Addo. Although the ward was closed after Dr Addo's death in 1999, the South AIDS Support, an NGO, campaigned to increase public awareness of HIV/AIDS and lobbied the South-West RHA to have the ward reopened. This was done in July 2002. The service has since expanded to include in-patient care and weekly outreach clinics in other areas of the country (SWRHA 2009).

### The prevention of mother-to-child transmission of HIV/AIDS programme

The government's prevention of mother-to-child transmission (PMTCT) programme began with the first pilot in July 1999 in Tobago, while the Trinidad pilot was initiated in 2000. These pilots were a direct result of the development of a national policy for the reduction of perinatal transmission of HIV by the Ministry of Health. By 2009, approximately 96.7% of all women attending public antenatal clinics throughout Trinidad and Tobago participated in the programme (NACC 2010a). Table 1 provides details of the number of women tested in 2008 and 2009.

**Table 1.** Pregnant women tested and results.

Category	2008	January to September 2009 (Trinidad only)
New pregnant women	15,963	12,059
No. of women first tested this pregnancy	15,625	11,662
Percentage of women tested	97.9	96.7
No. of women previously tested positive	94	NA
No. HIV-positive ELISA	123	73
No. of positive cases	217	130

*Source:* Adapted from *United Nations General Assembly Special Session on HIV/AIDS (UNGASS) Country Progress Report, Trinidad and Tobago* (NACC 2010a:28).

In 2008, dried blood spot testing for infants was introduced and in that year 98 infants were tested, with seven HIV-positive results. Of the 65 infants from January to September 2009, there were also seven positive results (NACC 2010a). Both the Eastern RHA (ERHA) and the SWRHA have formed support groups for mothers who are infected as well as families who are affected.

### Antiretroviral treatment and VCT

In October 2001, the Government of Trinidad and Tobago, with assistance from UNAIDS, successfully negotiated an 85–90% reduction in the cost of antiretroviral (ARV) drugs from four leading drug companies, namely, Merck Sharpe & Dohme, GlaxoSmithKline, Boehringer-Ingelheim and Bristol-Myers Squibb (Yearwood & Cumberbatch 2003). At the time of completion of the negotiations, the two main organizations in Trinidad and Tobago with experience in the use of these drugs were NGOs – the Medical Research Foundation (MRF) and the Cyril Ross Nursery (CRN).

ARV therapy (ART) is now available free of charge at the government hospitals and at seven locations throughout the country. At the end 2009, there were an estimated 3592 patients receiving ART with the major provider being the MRF (one of the seven sites), under agreement with the MoH. Other providers include the Scarborough Regional Hospital in Tobago and the Sangre Grande Hospital. Additionally, the Eric Williams Medical Sciences Complex and the CRN were the main providers of paediatric treatment and care having 79 and 78 patients, respectively (NACC 2010a).

Continuous training of the current pool of healthcare professionals is a major challenge, however, in order to maintain the service a reliable and widely available pool of trained staff will certainly be required.

Rapid (same-visit) testing and VCT can be accessed at 28 sites throughout the country. Although the National HIV Testing and Counselling Policy gained approval in 2009 and was widely circulated, many persons are still not aware of their HIV status. In 2009, 295 HIV-positive cases were identified out of a total of 15,685. Of those tests that were positive, 52.5% were female and 47.5% were male (NACC 2010a).

### Trinidad Public Health Laboratory and the National Surveillance Unit

All the HIV testing in the public sector is done by the Trinidad Public Health Laboratory (TPHL). Up to the early to mid-2000s, the Caribbean Epdemiology Centre (CAREC) provided all the confirmatory testing for the TPHL. However, the TPHL currently carries out the confirmatory tests for the public sector and also provides confirmatory testing services for some private sector laboratories (HEU 2012).

The National Surveillance Unit (NSU) of the TPHL has responsibility for the surveillance of HIV/AIDS in the country. The Unit is responsible for tracking the burden of the disease, and for detecting changes in the epidemic and AIDS-related morbidity and mortality. These data are obtained mainly from the public health sector, but also include other sources such as the National Blood Transfusion Service, QPCC&C, private laboratories, private doctors, and registries of births and deaths (HEU 2001). In order to address the challenges with respect to data collection and analysis, an information technology platform geared towards more efficient data processing has been set up at the NSU (NACC 2010a).

### Financing

Despite the involvement of the state, mainly through its Ministry of Health, up to the launch of the NSP, there had been no *major* expenditure reallocation and fiscal adjustments in the name of HIV/AIDS. Expenditure in the name of HIV/AIDS remained under one million dollars annually and in 1999 amounted to TT$850,000, 16 years into the epidemic. In 2001 expenditure slightly exceeded one million (TT$1.2 million) (Yearwood & Cumberbatch 2003).

**Fig. 1.**
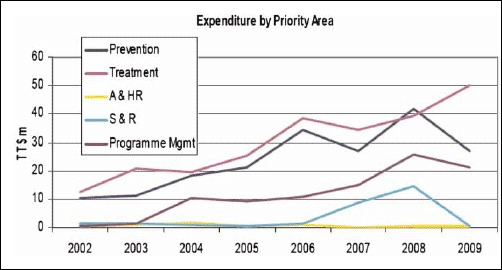
Expenditure on HIV/AIDS programmes 2002–2009. Reproduced from Draft Summary National HIV and AIDS Spending Assessment: An Assessment of HIV and AIDS Financing Flows and Expenditure 2002–2009 in Trinidad and Tobago (NACC 2010b:10).

In 2002, the government announced its intention to allocate TT$500 million to the fight against HIV/AIDS over the subsequent five-year period. Although there was a time lag, the NSP and the NACC were launched in March 2004. Figure 1 displays the trend in expenditure on HIV/AIDS between 2002 and 2009.

Between 2002 and 2009, there was an increase in total (capital and recurrent) expenditure from TT$25.2 million TT$98.95 million. Over the period, the total expended was TT$560 million. In 2009, the national recurrent budget for HIV was TT$39.2 million of which funding to the tune of TT$1.32 million was targeted to civil society organizations (CSOs). This was chiefly due to the increase in rapid testing facilities which grew from 5 in 2007 to 28 in 2008 (NACC 2010b). The year 2008 saw an all time high in the capital budget of TT$73.2 million, but by 2010 due to economic constraints the budgeted allocation fell to TT$41.8 million (NACC 2010b).

## Non-health public sector

### Social work

The government's commitment to provide for the welfare of citizens has been expressed explicitly through its undertaking of wide-ranging social service programmes. In spite of this however, there is a general inadequacy of the social support system to deal with many of the social problems, some of which are directly or indirectly linked to HIV/AIDS (child sexual abuse, street children, adolescent prostitution, gender-based violence, including rape).

In addition to the National Family Services Division, which has a staff of social workers, there are also school Guidance Officers. The social worker plays a key role in prevention, through working with different community teams in the delivery of information and education programmes to the society. Institutions that are targeted include schools (children in classrooms and parent-teacher meetings), health centre clinics, NGOs and the wider community. Most social workers give voluntary service to NGOs such as the Rape Crisis Society, the Child Welfare League and CARe, a support group for PLWHA.

The perception among many PLWHA is that the social services are often ad hoc and seriously under-resourced. However, in order to address the social problems comprehensively and to deal with the issues associated with HIV/AIDS, there is need for a vastly expanded cadre of social workers and counsellors.

### Public assistance

Public assistance is available to needy persons prevented by medical disability or other serious circumstance, from earning a living. Medical disability includes broken limbs and illnesses such as cardiac disease, end-stage renal failure and HIV/AIDS. Given their special nutritional and medical needs, many PLWHA who are not of independent means encounter problems meeting those needs. In addition, the need for housing in some cases as well as privacy and confidentiality when accessing the public assistance that is available make the need for reform all the more urgent. This is one challenge facing the public assistance and related-social sector agencies in Trinidad and Tobago today.

### Education of children with HIV/AIDS

Although the government's policy is that primary school education is compulsory and the constitution is intended to protect all citizens against discrimination, there have been instances of children who are HIV positive being refused admission into schools in Trinidad and Tobago. This is a violation of Article 28 of the United Nations Convention on the Rights of the Child, which the country has ratified. This ratification places an obligation on the government to address such discrimination in particular, and the overall stigmatization attached to HIV/AIDS.

There have been moves by the Ministry of Education and the Ministry of Health towards incorporating a Health and Family Life Education Programme in schools. Additionally, the Adolescent Mothers Programme of the Ministry of Social Development has a component that addresses awareness and education to the target group of children with HIV/AIDS. The Draft National Plan of Action for Children also addresses issues related to HIV/AIDS.

### Legislation

There have been three major initiatives with respect to HIV/AIDS-related legislation. The first was the Amendment to the Sexual Offences Act, 2000. This Act sought to provide monetary compensation for a complainant who contracted HIV from an accused person. The other was a draft Bill introduced in Parliament in October 2000, the Basic Conditions of Work and Minimum Wages Bill, which included provisions for persons with HIV/AIDS (La Foucade, Laptiste & Ashton 2003). The Bill contained important provisions for safeguarding the rights of PLWHA in the workplace, which included the following:

Employers shall not discriminate against an employee in hiring, firing and other terms and conditions of work and wages on grounds that the employee is infected with HIV and AIDS [*sic*].

An employer shall not require an employee to be screened or tested as evidence that the employee is not infected with the HIV virus and AIDS [*sic*], in circumstances where the information is not relevant to the type of employment offered to or performed by the employee. (Basic Conditions of Work and Minimum Wages Bill 2000)

Provision was also made for confidentiality; protection against stigmatization and discrimination by co-workers, unions, other employers or clients; for counselling or appropriate referrals; for reasonable alternative working arrangements; for protection against termination on the basis of HIV/AIDS and for information and educational programmes for all employees.

The Offences Against the Person (Amendment) (HIV) Bill was introduced in the House of Representatives on 29 October 2004. This Bill sought to amend the Offences Against the Persons Act, Chapter 11:08, in order to make it an offence to intentionally or recklessly expose another person to infection with the HIV. While there is every reason why the law should provide protection for uninfected persons, being infected with HIV should not cause any loss of equality before the law.

## The private health sector

### Services and facilities

The private health sector in Trinidad and Tobago has been a major provider of primary care health services and has emerged as a significant provider of secondary services. The sector became increasingly involved with HIV/AIDS prevention activities in private hospitals and clinics. One example is the Augustus Long Hospital, run by Petrotrin, a state-owned company. The hospital provides health education and health promotion and has a protocol in place for post-exposure prophylaxis (PEP) up to one month after exposure. A PMTCT Programme was introduced in 1998 at its antenatal clinics (ANCs) and, if found positive, mothers are offered ARV treatment for themselves and the unborn child.

### Diagnostic services

HIV/AIDS testing is available from three main private sector sources:

1. at the offices or walk-in clinics of private medical practitioners who utilize the Spot or Quick Tests to obtain results;
2. at private laboratories that conduct testing upon referral from a medical practitioner or private insurance company; and
3. at private hospitals.

It appears that unlike the situation in the public sector, private laboratories have no standard arrangements for confirmation of their screening tests. However, some do their confirmatory testing at the TPHL. This situation has serious implications for the efficiency of the national surveillance system, for when private sector data are not sent to the TPHL for confirmation, they do not enter the national surveillance network.

The current system of testing also raises several issues of equity. First, if private patients do not have the benefit of confirmation in each case, this subjects them to a greater risk of *false positive* and *false negative* results. Second, since testing in the private sector is in a large part conducted without pre- and post-test counselling (approximately 30% provide these services), the psychological costs associated with a positive result are perhaps far greater for a private patient than for one using the public system.

### Treatment

While a large percentage of the population (approximately 60%) uses the private sector for ambulatory services, private in-patient care is too expensive for the average client, unless he/she has private health insurance coverage. In cases where the client requires admission, definitive statements cannot be made about the willingness or ability of the private sector to accept HIV-positive in-patient except to say that they are dealt with on a case-by-case basis.

Drugs for the treatment of opportunistic diseases related to HIV/AIDS are available through the private pharmacies when prescribed by the medical practitioner; however, private pharmacies do not routinely stock ART drugs.

### Assessment of private sector services

According to HEU (2001), there are many ‘unregulated’ laboratories, of varying quality, performing HIV screening tests in greater numbers than the public laboratories. By 2010, counselling services associated with HIV testing were not normally provided. Essentially, patients use general practitioners (over 450) and private hospital services to access care on a ‘fee for services’ basis, accessing the testing facilities but are unable to receive associated counselling and support services.

## International agencies

Several international agencies such as the PAHO, the International Labour Organization (ILO), the United Nations Population Fund, the UNAIDS, the European Union (EU) and the United Nations Development Programme (UNDP), among others, form part of the landscape of the social sector response to HIV/AIDS. It is of note that both PAHO and UNAIDS were actively involved with the Ministries of Health in the Caribbean region in negotiating for substantially reduced prices of ARVs in 2002. These drugs became one of the main components in the arsenal of weapons to fight the HIV virus and their availability resulted in a significant decrease in AIDS deaths as well as improved quality of life for PLWHA.

The UNDP, in particular, is involved in efforts to mitigate the social, economic and human developmental repercussions of the HIV/AIDS epidemic. Its programmes place heavy emphasis on capacity building of both countries and organizations, to enable them to adequately respond to the epidemic.

Similarly, UNAIDS, whose mandate is to facilitate the country's expanded response to the epidemic, played a key role in the development of several response programmes since its presence in the country from 1999 (UNTT 2009). UNAIDS functions under the umbrella of the UN Theme Group, which in Trinidad and Tobago includes NGOs such as the Community Advisory Board, CARe, Trinidad and Tobago HIV/AIDS Alliance and Artists against AIDS (HEU 2001). The agency provides funding for key areas of intervention in country programmes, especially those for vulnerable groups, PLWHA and youth and has been a major catalyst that drove the effort to the drafting and costing of the first strategic plan for HIV/AIDS for the country. UNAIDS also has a presence on Pan Caribbean Partnership Against HIV/AIDS (PANCAP), which coordinates response programmes at the regional level for Caribbean Community (CARICOM) member states.

The EU has been a key player in the social sector and signed a ‘Country Strategy’ with the Government of Trinidad and Tobago involving a ζ17.9 million co-operation programme for the period 2002–2007. An allocation of 80% of the resources was intended to cover the long-term development activities in the sector of tertiary education and 20% in health, more importantly for HIV/AIDS prevention. As a result of these efforts, some of the tertiary level personnel who benefitted from the programme were actively involved in crafting the strategic response, the development of the NACC as well as other initiatives.

Also involved in the response to the epidemic has been the World Bank, which in 2003, approved a US$20 million loan for programmes aimed at providing treatment and care for PLWHA and reducing HIV infections. The Clinton Foundation and the United States Agency for International Development (USAID) have assisted in the area of capacity building for the clinical management of patients with HIV/AIDS.

The ILO provided critical support for the development of a National Workplace Policy on HIV/AIDS (NACC 2010a). In 2007, in collaboration with the NACC, the ILO hosted a training workshop on HIV/AIDS and Rights at Work and in April 2008, the National Workplace Policy on HIV/AIDS was launched (NACC 2010a). This was indeed a significant milestone in the response to HIV/AIDS landscape.

## Non-governmental, community-based and faith-based organizations

In the area of NGO participation in HIV/AIDS programmes, the need for coordination of activities is one of the main challenges. An umbrella organization, the Trinidad and Tobago HIV/AIDS Alliance, was established to address this issue. Several voluntary organizations that provide some degree of service to PLWHA are part of the alliance. They can be categorized as follows:

- Care and Support Groups;
- Faith-Based Organizations;
- Human Rights, Advocacy & at Risk Support Groups;
- Medical Community-Based Organizations;
- National Agencies;
- Reproductive Health and Rights Groups;
- Women's Organizations; and
- Youth Organizations.

Some organizations keep a low profile because of homophobia and the discrimination and stigma associated with the disease. Other groups that provide various forms of support for PLWHA may not be officially recognized, nor are their efforts limited only to PLWHA. One example of this is Petals Foundation, which provides education and recreational programmes for children in the smaller care homes. Some of the organizations and services listed above operate outside of the framework of the state, whilst many are linked to the state through core financing and subventions, policy agreements and contracts. All however, must comply with the legislative and policy requirements established by government bodies. Some of the key NGO/CBO/Faith-based Organization (FBO) agencies are highlighted below.

### Medical Research Foundation

Prior to the introduction of the government's PMTCT programme, the MRF – an independent research institution headed by Professor Courtenay Bartholomew – initiated a PMTCT programme in June 1997 (Yearwood & Cumberbatch 2003). Working closely with the Ministry of Health, the MRF offered Azidothymidine (AZT) to pregnant women living with HIV. The programme was enhanced in 1998 and the treatment of the newborn was included. This programme was well accepted by many HIV-positive women who received AZT prenatally for one to four weeks, followed by treatment of the newborn for six weeks.

The MRF has also led to the development of the medical research programme in HIV/AIDS, conducting collaborative research studies with the National Cancer Institute and the National Institutes of Health, Maryland, USA. The Foundation was also responsible for the conducting of the HIV vaccine trials being carried out in Trinidad and Tobago.

### CARe

Providing emotional support to the PLWHA, CARe is a non-profit organization run by PLWHA. The organization offers professional counselling for both individuals and groups, and provides to its clients critical information on the management of HIV/AIDS, understanding medical terms, and other daily issues facing PLWHAs. The overarching goal of CARe is to empower PLWHA and improve their quality of life. Like other NGOs, CARe interacts closely with the Caribbean Regional Network of People Living with HIV/AIDS (CRN+), which was established in 1996.

The PLWHA community has been very much involved in the response to HIV/AIDS, in the face of extreme odds. In June 2004, the CRN+ was awarded US$3 million in grant funding from the Global Fund to continue its work in the region and build the institutional capacity of local networks such as CARe.

### Tobago Oasis Foundation

Established in November 2000, the Tobago Oasis Foundation provides services to PLWHA. The aim of the Foundation is to promote the welfare and dignity of persons with HIV/AIDS. This is done through a range of programmes involving treatment and care, prevention, health, education and spirituality, with a full awareness of the cultural traditions of the people. Some of the programmes include outreach to PLWHA and their families, working against stigma and discrimination, as well as fostering regional and international networking.

### Tobago AIDS Society

This Society was founded in 1996 and has as one of its main objectives, assisting PLWHAs with the purchasing of drugs for the opportunistic diseases that result from HIV/AIDS. The Society also helps PLWHAs and their families with financial assistance for housing when they become homeless, and also gives support for school fees, uniforms, books and food to children whose parents are living with HIV, who may themselves be living with HIV or who are orphans. Home care is also provided for those who are ill.

### Arts in action

The role of culture in the communication of health messages is critical. The Grassroots group such as Arts in Action has been involved in health education and promotion workshops for children aged 6 – 12 years, teenagers at secondary school and youth and adults in tertiary education, in the workplace and in communities (Arts in Action 2002). Established since 1994, the group employs interactive educational theatre with their audiences. The group's ‘Jus’ Once' project highlights seven stories that are told by young people concerning their personal experiences with HIV/AIDS. These are shared with the participating audience and linked by music and song and are opened up for discussion and suggestions from the audience by facilitators who hot seat and role play the characters seeking solutions and options through positive intervention and knowledge. In 2004 this project was recognized as one of the top ten ‘Techniques and Practices for Local Responses to HIV/AIDS’ by the UNAIDS and the United Nations Institute for Training and Research (UNITAR) (culturebase.net 2007).

### The Toco Foundation

The Toco Foundation, located in the north-east region of Trinidad, is a grassroots community enterprise that has achieved astounding success in its short existence. Established in 1994, the Foundation's activities are separated into three distinct units to streamline operations and make service delivery more efficient. The units are Communications, Social Services and Agro-Tourism.

With the possible exception of Radio Toco, the Toco Youth Sexuality Project (TYSP), run by the Social Services Unit, is perhaps the most well known of the projects undertaken by the Toco Foundation. This project was awarded the Commonwealth Award, first place, for Youth and HIV/AIDS, 2002. The TYSP works with two thrusts. The in-school unit visits schools along the coast from Matura to Matelot, a distance of 38 miles (and goes to other counties if requested). The out-of-school arm visits prenatal and post-natal clinics, youth groups, village councils, church groups and popular liming spots; it also does house-to-house education. Both arms deliver a powerful message on all the issues surrounding HIV/AIDS, sexuality and STIs, establishing the facts and clearing up the myths that exist. They also cover topics like negotiation skills, nutrition and hygiene. These visits make use of dramatic presentations, role-playing techniques, interactive talks and condom demonstrations to deliver the message to the audiences.

Presentations are also made via Radio Toco. On these programmes presentations are arranged thematically and the issues that are presented during the face-to-face sessions are covered in great depth; time is devoted to listeners who either call in or mail in their questions. From time to time, a forum is also given to presenters outside the TYSP who do work in the field to discuss their efforts and to answer questions.

### Cyril Ross Nursery

The CRN, a residential operated by the Society of St. Vincent De Paul, was opened in September 1994. It is the major FBO involved in the treatment and care of PLWHA. The Nursery cares for children with HIV/AIDS and is the first and only facility in the country to specialize in caring for children living with the virus. As its name suggests, it was originally intended for very young children, since prognosis for the disease at that time indicated such a shortened life span, that children were not expected to survive much beyond early childhood. The facility was originally designed to cater for 25 children, but has 35 children in its care, ranging from 1 to 18 years of age.

Children are referred to the Nursery by Medical Social Workers who are in constant contact with hospitals, where babies born with HIV are sometimes abandoned by their parents. Children whose parents have died from AIDS and those whose parents are unable to provide adequate care for them are also admitted to the Nursery. The Nursery gets a subvention each year from the Ministry of Social Development and has also been a recipient of a number of grants from the NACC.

The Nursery has a school within its compound that caters to the educational needs of the younger children. Most of the older children attend St. Mary's Anglican Primary School. A doctor provides his services free of charge and pays regular visits to the Nursery. The children also receive paediatric care at the Eric Williams Medical Sciences Complex. There is no social worker at the Nursery and social work services have been provided through the placement of students from the Social Work Unit of the University of the West Indies.

This model can be duplicated in communities throughout Trinidad and Tobago, with NGOs providing the emotional and physical support needed by children living with HIV. However, it must be noted that homes like these were initially meant to be temporary, with the aim of reintroducing the children into their families and communities. To the extent that this aim is not accomplished, there is need to ensure that children will be cared for in familiar surroundings, attend district schools and churches and take part in local recreational activities, so that communities can become involved in their development.

## Workplace

### National workplace policy

A National Policy on HIV/AIDS in the Workplace had its genesis in a National Consultation on HIV/AIDS in 1992 and benefited from dialogue among a varied cross-section of the society – business professionals, health experts, NGO representatives and the AIDS Programme Co-ordinator (HEU 2001). The Workplace Policy on HIV/AIDS was eventually approved and launched in April 2008 with the support of the ILO (NACC 2010a).

Additionally, a post-exposure policy for healthcare workers has existed since 2000. The policy involves protocols for the use of ART, rapid HIV laboratory testing and counselling.

### Trade unions

The trade union movement has been relatively low-keyed in its response. A representative from one of the leading trade unions indicated that the union had participated in seminars where discussions were held on HIV/AIDS in the workplace, but had not on its own, initiated programmes for its membership. According to the Situation and Response Analysis of HIV/AIDS in Trinidad, 2001:

*Three areas were identified under which issues around HIV/AIDS can be addressed. These were through Occupational Health and Safety (OHS) programmes, Employees Assistance Programmes (EAP) and programmes geared to promote healthy lifestyles. It was felt that of the three, either the EAP or general wellness programme was better placed to address the issue of HIV/AIDS. It was not clear however, that the latter was as widely implemented as the former. The focus of the OHS programme has tended* historically to focus on the areas around accidents and safety and less on general health components. *(HEU 2001:64)*

The trade union movement has begun making small steps towards dealing with the issue of HIV/AIDS. One of the reasons put forward for the past lack of commitment is that persons have not come forward stating that they have been discriminated against in the workplace as a result of their positive HIV/AIDS status. Union officials indicated that it is difficult for trade unions to accomplish change in organizational policies unless they have the openings of negotiating collective agreements. It is common practice now that in every new agreement negotiated by the union, a clause is inserted against discrimination against PLWHA.

Another large union, the Public Services Association (PSA), commissioned all its members with direct links to PLWHA and whose work entails dealing ‘hands on’ with HIV/AIDS – nurses, mental health workers, etc. – to formulate an internal policy for the Association. The union also provides monthly financial support to the South AIDS Support (SAS), which is a PLWHA support group. The SAS, in turn, does sensitization lectures for PSA members.

## Insurance industry

Insurance companies offer no services to persons known to be PLWHA, even though they may be long-standing customers. Often, the HIV test is done on clients submitting to medical tests for insurance without their knowledge and with no prior counselling.

The United Nations Development Programme (2003) argues for insurance companies to give incentives and discounts to companies with strong workplace and community prevention programmes. In Trinidad and Tobago, in contrast, there may be need for incentives to be *given to* insurance companies that do not discriminate against PLWHA. Further, in the face of reduced costs for, and improved access to, treatment for HIV/AIDS, as well as increased longevity associated with early treatment, insurance companies are presented with alternatives for coverage of PLWHA.

## Assessing the overall response

In assessing the impact of the response, the status of the epidemic points to some measure of success, especially in the area of treatment. The data indicate that there has been a small but continuous increase in HIV prevalence rates from 1.2% to 1.5% between 2006 and 2009 (NACC 2010a). With the expansion of access to ARVs, a programme that began in 2002, persons are living longer and healthier lives with the disease and there have been annual reductions in the number of reported deaths from AIDS. There was a decline in mortality from 114 in 2007 to 72 in 2010 (Republic of Trinidad and Tobago 2013). The increase in the number of sites offering same-day testing as well as improvements in data collection and analysis have also contributed to the reports of increased prevalence rates.

The number of new infections reached a high of 1709 in 2003 (NACC 2010a) but has since then fallen in every subsequent year, numbering 1154 in 2010 (Republic of Trinidad and Tobago 2013). This indicates that prevention programmes have had some positive outcomes (Table 2).

**Table 2.** Cumulative HIV/AIDS cases and deaths 1983–2010

	2007	2008	2009	2010	Cumulative Total 1983–2010
New HIVpositive	1429	1448	1390	1154	22,829
AIDS	163	97	124	72	6407
Deaths	114	87	77	72	3999

*Source*: Adapted from *Republic of Trinidad and Tobago National HIV and AIDS Strategic Plan (2013–2018)*, p. 15.

The PMTCT programme is one of the more successful prevention strategies. An increasing number of antenatal clinic attendees are tested nationwide and in both Tobago and the county of Caroni-North, there has been the achievement of 100% testing of clinic attendees. Despite challenges such as inadequate follow up of mothers and babies, access to all babies who need to be tested and lack of testing of partners and other children, the programme has had a positive impact on reducing the transmission rates from mothers to their children.

It would also be useful to contextualize the response based on the Millennium Development Goal indicators and those of (UNAIDS 2002, 2009; WHO 2004a, 2004b) that were developed for that purpose. As seen in Table 3, the country scores very well in the areas of Strategic Planning, Care and Support and Prevention. Overall, the laws of the country do not specifically provide protection against discrimination on the ground of HIV status or suspected HIV status. Although the Equal Opportunity Act 2000 exists it addresses general anti-discrimination, but not HIV directly. There is need however, to look at the rate of progress in adopting legislation that will protect the rights of PLWHA. In 2010, the NACC had undertaken a legislative assessment along with a draft national policy with a view towards developing legislation on HIV to address stigma and discrimination issues (NACC 2010a). Additionally, given the preceding discussions on the involvement of the various sectors of the society, there is also need to fill in gaps, especially as they pertain to social services for PLWHA in general and children in particular.

**Table 3.** National commitment and action: millennium development goal indicators.

	Status in Trinidad and Tobago		
	Yes	No	NA
A. Strategic plan	✓		
1. Country has developed multisectoral strategies to combat HIV/AIDS	✓		
2. Country has integrated HIV/AIDS into its general development plan	✓		
3. Country has a functional national multisectoral HIV/AIDS management/coordination body	✓		
4. Country has a functional national HIV/AIDS body that promotes interaction among government, the private sector and civil society	✓		
5. Country has a functional HIV/AIDS body that assists in the coordination of civil society organizations	✓		
6. Country has evaluated the impact of HIV/AIDS on its socioeconomic status for planning purposes	✓		
7. Country has a strategy that addresses HIV/AIDS issues among its national uniformed services (including armed forces and civil defence forces)	Part of overall strategy		
B. Prevention			
1. Country has a general policy or strategy to promote IEC on HIV/AIDS	✓		
2. Country has a policy or strategy promoting reproductive and sexual health education for young people	✓		
3. Country has a policy or strategy that promotes IEC and other health interventions for groups with high or increasing rates of HIV infection	✓		
4. Country has a policy or strategy that promotes IEC and other health interventions for cross-border migrants			✓
5. Country has a policy or strategy to expand access, including among vulnerable groups, to essential preventative commodities	✓		
6. Country has a policy or strategy to reduce MTCT	✓		
C. Human rights			
1. Country has laws and regulations that protect against discrimination of people living with HIV/AIDS			Laws under assessment
2. Country has laws and regulations that protect against discrimination of groups of people identified as being especially vulnerable to HIV/AIDS			Laws under assessment
3. Country has a policy to ensure equal access for men and women to prevention and care, with emphasis on vulnerable groups			Laws under assessment
4. Country has a policy to ensure that HIV/AIDS research protocols involving human subjects are reviewed and approved by an ethics committee	✓		
D. Care and support			
1. Country has a policy or strategy to promote comprehensive HIV/AIDS care and support with emphasis on vulnerable groups	✓		
2. Country has a policy or strategy to ensure or improve access to HIV/AIDS-related medicines, with emphasis on vulnerable groups	✓		
3. Country has a policy or strategy to address the additional needs of orphans and other vulnerable children		Under review	

*Source:* Adapted from *Declaration of Commitment on HIV/AIDS: Core Indicators' Revision* (UNAIDS 2002:5–13). http://data.unaids.org/Topics/M-E/merg8_ungass-core-indicators_en.pdf

## Conclusion: connecting the pieces of the response quilt

Since HIV/AIDS affects all levels of humanity, whether as individuals, families, communities and countries, in our increasingly interdependent world, no class is immune from this epidemic. The NSP, which provides the blueprint for the coordinating function details in large measure, this very interconnectedness and the roles of the various agencies. Table 4 provides some measure of the length and breadth of the quilt, and some of the relationships that need to be fostered. The relationships presented here are by no means exhaustive, but give an indication of the necessary interactions between different agencies if the expanded response is to be successful.

**Table 4.** The HIV/AIDS expanded response quilt.

	Government	Workplace	Community	Individuals
Government	Public agency arrangements:	Opportunity for public/private partnering	Capacity building	Heeding programme messages
	Blood supply Hospital care Laboratories Surveillance	Facilitating comprehensive response, technically and financially	No tax evasion
Workplace	Legislation pending	Workplace policy approved and launched	Continued technical support and moral pressure to ensure adherence to workplace policy	Caring relations with infected colleagues
Community (including international organizations)	Public education QPCC&C MRF – PMTCT/ARV	Opportunity to focus on vulnerable groups	Spawning and strengthening of NGOs, particularly for PLWHAs	Voluntary service on behalf of PLWHAs
Individuals	Access to care	Opportunity for frontal attack on stigma and discrimination issues;	Working to reduce stigma and discrimination	Family support
	Quality of caring	looking after affected persons	NGO support for care and adherence	Responsibility to partner
	Public assistance			Choosing safe practices

Although countries look to international experience to help in fashioning their response, the nature of the epidemic demands that actions be taken by the individuals, organizations, groups and communities *within* each county. These national groups have contributed greatly to the response, and their efforts should continue unabated.

From the experience of other countries, we may identify the ingredients for a successful response as follows:

(1) *Clear understanding by the community and the leaders of the nature of the phenomenon.* This presumes that there is ample evidence/research, and that there is an effective machinery for disseminating information.
(2) *Appropriate technical inputs.* This includes the identification of proper instruments and resource requirements, inclusive of existing agencies or institutions and policies/programmes.
(3) *A general commitment to initiate and sustain the response on a personal, social or community and political level.* The needed adjustments in time and resources will depend on this commitment.

Given the reality of objectively determined obstacles to the response, including a shortage of trained personnel in many areas and information systems in need of modernization, it becomes even more important that in Trinidad and Tobago, a conscious effort be made to speed up the implementation of measures to continue the fight against the spread of HIV/AIDS.
